# Study protocol of a randomized controlled trial on two new dissemination strategies for a brief, shared-decision-making (SDM) training for oncologists: web-based interactive SDM online-training versus individualized context-based SDM face-to-face training

**DOI:** 10.1186/s13063-018-3112-7

**Published:** 2019-01-07

**Authors:** Nicole Müller, Kathrin M. Gschwendtner, Sarah Dwinger, Corinna Bergelt, Wolfgang Eich, Martin Härter, Christiane Bieber

**Affiliations:** 1 Department of General Internal Medicine and Psychosomatics, Center for Psychosocial Medicine, Heidelberg University Hospital, Thibautstraße 4, 69115 Heidelberg, Germany; 2 Department of Medical Psychology, Center for Psychosocial Medicine, University Medical Center Hamburg-Eppendorf, Martinistr. 52, 20246 Hamburg, Germany

**Keywords:** Shared decision-making, Randomized controlled trial, Oncology, Web-based online training, Coaching, Standardized patients

## Abstract

**Background:**

Oncological patients often feel left out of important treatment decisions. However, when physicians engage them in shared decision-making (SDM), patients benefit in many ways and the situation is improved. SDM can effectively be taught to physicians, but participation barriers for SDM physician group trainings are high, making it hard to convince physicians to participate.

With this in mind, we aim to develop and evaluate two new dissemination strategies for a brief, SDM training program based upon a proven SDM group-training concept: an individualized context-based SDM face-to-face training (IG I) and a web-based interactive SDM online training (IG II).

We aim to analyze which improvements can be achieved by IG I and II compared to a control group (CG) in physician SDM competence and performance as well as the impact on the physician-patient relationship. Furthermore, we analyze differences in satisfaction concerning the two dissemination strategies by means of a training evaluation.

**Methods/design:**

We examine – based on a three-armed randomized controlled trial (IG I, IG II, CG) – the effectiveness of two new dissemination strategies for a SDM training program compared to a CG receiving no SDM training (voluntary access to SDM training as an incentive for participation after completion of the study). We aim to include 162 physicians randomized to one of the three arms. There will be two assessment points in time (before intervention: T_0_ and post-training: T_1_). The main outcome is the SDM competence of physicians as measured by an established observational assessment rating system (OPTION-12) by means of consultations with Standardized Patients. Standardized Patients are individuals trained to act as “real” patients. Secondary outcome measures are the SDM performance (SDM-Q-9) and the Questionnaire on the Quality of Physician-Patient-Interaction (QQPPI) both rated by Standardized Patients as well as the physicians’ training evaluation.

**Discussion:**

This trial will assess the effectiveness and acceptability of two new dissemination strategies for a brief, SDM training program for physicians. Opportunities and challenges regarding implementation in daily routines will be discussed.

**Trial registration:**

ClinicalTrials.gov, Identifier: NCT02674360. Prospectively registered on 4 February 2016.

**Electronic supplementary material:**

The online version of this article (10.1186/s13063-018-3112-7) contains supplementary material, which is available to authorized users.

## Background and rationale

Different studies have revealed that cancer patients do not feel sufficiently involved in important treatment decisions [1, 2]. Patients often feel that physicians decide in a too paternalistic way and do not consider the patient’s personal life situation, expectations, preferences and fears. Patients who experienced paternalism were less satisfied with the consultation and emotional support [3].

An attempt to overcome those shortcomings in patient-physician communication is the implementation of the model of shared decision-making (SDM) into routine care. SDM is seen as an ideal model for how physicians and patients should interact in medical decision-making [4]. In this concept, both the patient’s medical and personal information are shared between physician and patient, and both parties (in an equal position) subsequently come to a shared decision as a team. Implementation of SDM requires that the physician has a patient-centered attitude and is open to the needs of the patient.

A review by Chewning et al. [5] about role preferences in medical decision-making showed a trend to an increasing demand for SDM by patients in recent years. Studies indicate that if SDM is implemented, patients show a higher level of treatment satisfaction [6–8], a higher level of risk comprehension, less decisional conflict [9], more realistic expectations towards the therapy [10], a higher level of treatment adherence and better coping with illness [11]. Furthermore, a higher quality of physician-patient interaction can be achieved [12].

Despite physicians’ positive attitude towards SDM [13], implementation in daily practice is insufficient (e.g., [14, 15]), as there are many barriers such as time constraints, patient characteristics and the nature of the clinical situation [16].

In recent years, there has been a growing number of SDM training programs for physicians [17] which teach them how to involve their patients in important treatment decisions [18, 19]. However, it remains unclear what kind of intervention is most effective to enhance physician competence at SDM [20].

Earlier studies have shown that it is difficult to enroll physicians for an SDM training intervention that occurs in an external setting. Especially, economic and psychological factors seem to represent the major barriers [18]. Moreover, physicians frequently overestimate their own communication skills [21], which consequently leads to an unwillingness to spend time participating in communication trainings.

Because of these circumstances, it has been suggested that new SDM training strategies might be necessary that can be administered independently of time and place: e.g., individualized, context-based, face-to-face SDM trainings or web-based online SDM trainings constitute possible alternatives for common SDM group-based trainings [18, 22] and could potentially address physicians’ temporal and locational constraints that have previously inhibited participation.

Context-based communication trainings for physicians have achieved good results in the improvement of physicians’ communication skills in clinical encounters [21, 23, 24]. Moreover, from the physicians’ point of view, face-to-face interventions have some advantages: They feel less undermined in their expert role, and their existent communication skills are recognized [21].

Web-based trainings are effective opportunities for physician education [25–28]. The challenge in developing SDM online trainings consists in providing practical (procedural) and not merely factual (declarative) knowledge [25, 29, 30]. There is evidence that well-designed online tutorials facilitate the acquisition of complex skills as well as reduce time in the acquisition of these skills compared to traditional formats such as personal lectures (e.g., [31]). Well-designed online trainings show advantages regarding time efficacy and memory effects compared to traditional teaching methods such as frontal presentations [32, 33], which seem to be effective in improving knowledge but less effective in changing behavior [34, 35].

However, when compared to web-based trainings, face-to-face trainings generally seem to be more effective [26].

This study protocol was written in accordance with the Standard Protocol Items: Recommendations for Interventional Trials (SPIRIT) guidelines. The SPIRIT Checklist has been included in Additional file 1.

### Objectives

The current study aims to develop and evaluate two innovative dissemination strategies for a brief, SDM training program for physicians. Based on an established and evaluated comprehensive SDM group training concept [18], we adapt an individualized, context-based, SDM, face-to-face training (intervention group I: IG I) and a web-based interactive SDM online training (intervention group II: IG II). We aim to evaluate which improvements in physician SDM competence and performance can be achieved by these two interventions compared to a control group (CG) without SDM training and if the quality of physician-patient interaction is influenced. SDM competence and performance as well as physician-patient interaction are assessed in consultations with Standardized Patients (SPs) before and after the intervention. SPs, also known as simulated patients, are individuals trained to act as “real” patients in order to simulate a set of symptoms or problems in a clinical scenario in a standardized way, allowing direct comparison of the physicians’ SDM and communication skills.

We derive the following hypotheses:

#### Main hypothesis

##### Hypothesis 1

We hypothesize that in both intervention groups (IG I and II), physicians will show a higher level of external-rated SDM competence (assessed by OPTION-12 [36–38]; adjusted for T_0_) during their consultation with an SP 4 weeks after the training session (T_1_) compared to a CG (no training).

#### Secondary hypotheses

##### Hypothesis 2

In both intervention groups (IG I and II), physicians will show a higher level of SDM performance (assessed by SDM-Q-9 [39, 40]; adjusted for T_0_) rated by an SP during consultation 4 weeks after the training session (T_1_) compared to physicians in the CG (no training).

##### Hypothesis 3

In both intervention groups (IG I and II), physicians will show a higher level of quality in their physician-patient interaction (assessed by the Questionnaire on the Quality of Physician–Patient Interaction (QQPPI) [41]; adjusted for T_0_) rated by an SP during consultation 4 weeks after the training session (T_1_) compared to physicians in the CG (no training).

##### Hypothesis 4

Physicians receiving the individualized context-based SDM face-to-face training (IG I) will be more satisfied with the training (assessed by self-developed training evaluation) compared to physicians receiving the web-based interactive SDM online training (IG II).

### Trial design

**Fig. 1:**
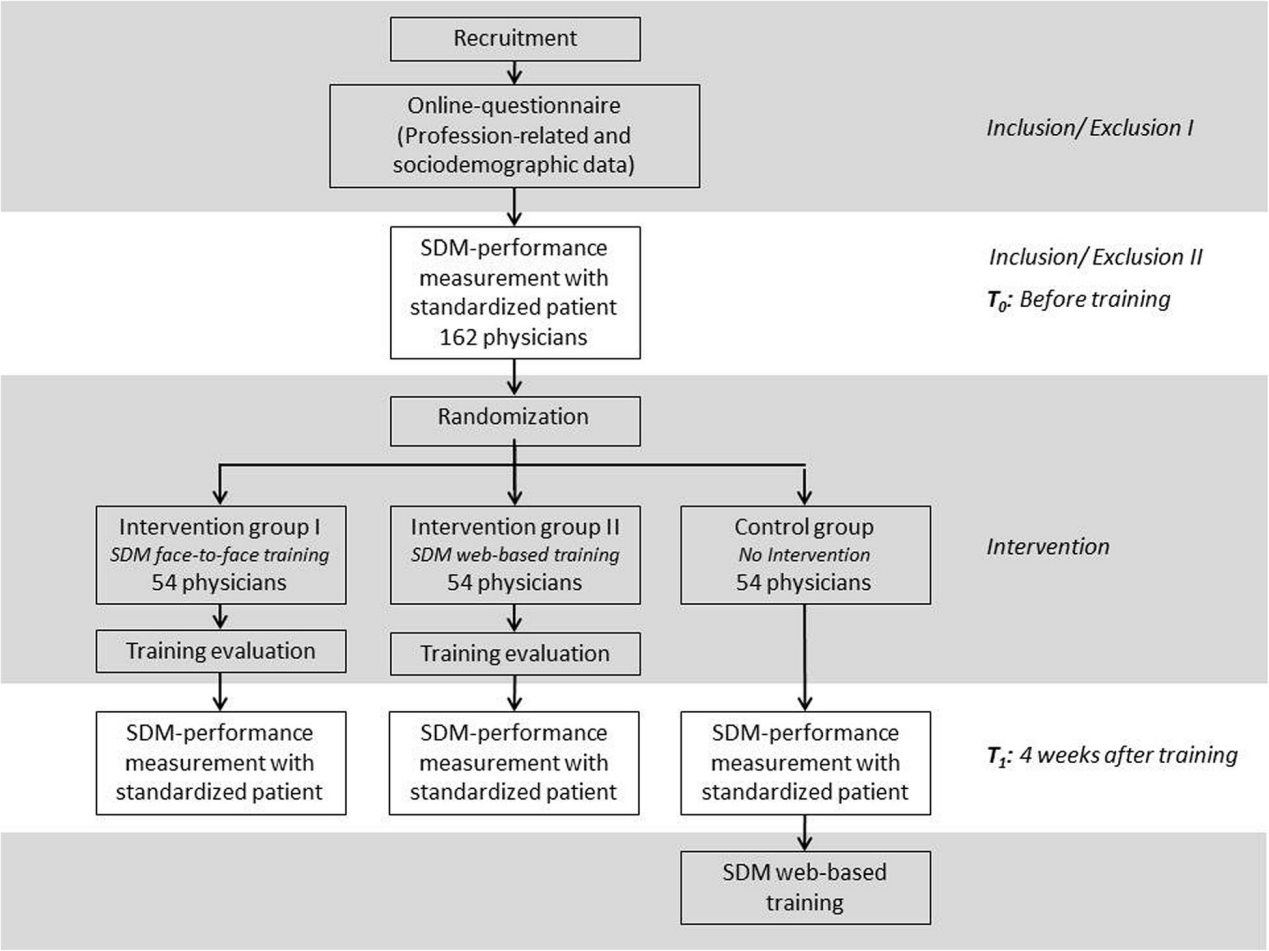
Study design

The study uses a bicentric, three-armed, randomized controlled, pre-post design. We will include 162 physicians who work either with colon cancer patients (stage II with risk factors) or breast cancer patients (stage II). In these two stages of disease patients are confronted with highly preference-sensitive decisions: For colon cancer stage II with risk factors (e.g., after an emergency surgery, perforation of the tumor, poorly differentiated tumor tissue) the German medical guidelines [42] recommend the use of SDM concerning the decision for or against chemotherapy after the surgical excision of the tumor. For stage II breast cancer the German medical guidelines [43] recommend SDM to choose between two possible options: breast-conserving surgery followed by obligatory postoperative radiotherapy or, alternatively, a complete removal of the breast. In each of the two IGs and in the CG, we aim to include 54 physicians. The study design and participant timeline are shown in detail in Fig. 1.

## Methods: Participants, interventions, and outcomes

### Study setting

Both study centers are responsible for physician recruitment and administration of SDM trainings; the University Medical Center of Heidelberg (HD) is responsible for the southern part of Germany, and the University Medical Center Hamburg-Eppendorf (HH) is responsible for the northern part. Physicians from many different backgrounds are included, assuming that they meet the eligibility criteria.

### Eligibility criteria

Inclusion/exclusion I: Physicians who work with colon or breast cancer patients will be included in the study. Furthermore, physicians are required to have Internet access, to sign a letter of consent for participating in the study and to agree to videotape their consultations with the SPs. Physicians who are not attending to oncological patients will be excluded from the study.

Inclusion/exclusion II: Physicians who fill out the online questionnaire for inclusion, but then do not conduct the consultation at T_0_ are defined as withdrawal. Withdrawal physicians are compared regarding sex, medical specialty, and years of professional experience with those participants who conduct consultations at T_0_ or T_0_ and T_1._ Those comparisons will be done using *t* and chi^2^ tests to identify a potential systematic withdrawal. Withdrawal physicians will not be analyzed further and will be excluded.

### Interventions

The intervention consists of a brief, SDM training program for physicians, either in the form of an individualized, context-based SDM face-to-face training in the physician’s office (IG I) or as an interactive, web-based, SDM online training (IG II). The CG of physicians receives no SDM training during the course of the study, but is granted voluntary access to SDM online training as an incentive for participation after completion of T_1_.

The content of both trainings (IG I and II) follows a comprehensive SDM training program previously developed and evaluated by the same study group [44]. It has recently been applied by other study groups [45]. However, in the present study SDM training content is shortened, condensed and adapted to the new dissemination methods.

Content relies on SDM and patient-centered communication. The learning objectives for participating physicians are:

- Knowledge of the theoretical basis of SDM
- Recognizing the participation needs of their patients
- Knowledge of techniques for an adequate transfer of information, risk communication, and performance of SDM during consultations
- Consultation structuring skills

Both new training strategies are interactive in character and participants are actively taking part. For instance, physicians are asked to define their own goals and the competences they want to achieve by the training. Also feedback is a fundamental component of both training strategies, enabling participants to constantly compare their aims and their current position [46], either by feedback given by the coach or with a self-rating using a reliable instrument (OPTION-12, German version; [36]).

The difference between the two training strategies is the method of the teaching procedures. In both IGs, the trainings have the same length (approximately 1.5 h). The trainings within the study are being offered free of charge for all participating physicians (including CG).

We evaluate the treatment integrity for both training strategies using an online survey (10 general items appropriate to both implementation strategies and four items specific for the web-based training or resp. five items specific for the face-to-face training. All specific items pertain either to specific interventions, exercises, work atmosphere, trainers' responsiveness or design of the two different training strategies.) send to the participants of the IG I and II after the training but prior to the second consultation with the SP at T_1_. The online survey assesses physicians’ satisfaction with content and realization of the trainings. In addition, in IG II, the evaluation of the user profiles (logfiles) will serve to assess adherence to the web-based training in IG II.

#### Face-to-face-training (IG I)

The individualized, context-based, face-to-face training follows the coaching principle. Participants receive the coaching module at their workplace with a coach/trainer from the project team. Prior to face-to-face training, the coach assesses the videotaped consultation with the first SP. During the face-to-face training, the coach provides feedback, reminds the participant of possible missed steps during his videotaped consultation and, if needed, demonstrates specific SDM steps. Favorable behavior is marked and reinforced and improvable behavior suggested and practiced. Finally, individual goals for improvement and practice are agreed on and fixed. Additionally, participants receive a booklet that they may keep, summarizing all relevant information on SDM. Two weeks later, the participating physician receives a booster session via telephone from their coach (maximum 30 min). In this booster session, it is assessed how the participant was able to put SDM into action, and the remaining questions and personal SDM-related goals of the participant are discussed.

The quality and consistency of the face-to-face training will be ensured through the regular internal supervision and training of participating SDM coaches/trainers. After each face-to-face intervention, the trainer completes a protocol of the intervention.

#### Web-based training (IG II)

The SDM online training is based on the modeling principle. Participating physicians learn by watching educational video clips in which SDM is performed to a very high standard by lay actors, namely, a very experienced senior physician functioning as a role model (i.e., head of the Breast Center) and a SP. This helps participating physicians develop their own mental model of the essential SDM steps and subsequently utilize them. In technical terms, the online training is conceptualized as a tutorial program [47]. The sequence is identical for every unit: introduction, presentation of the information, tasks, analysis of the answers, feedback, and conclusion of the unit [48]. There is no rigid order; each participating physician can adapt the individual units to his own needs. After watching his own videotaped consultation with a SP (DVD sent to the participant before), the physician is subsequently asked to rate his own performance by the OPTION-12 rating system (identical to the rating system the external raters use). Unit-related tasks in different formats (e.g., multiple choice questions and open questions) serve to underpin the intended learning process. One objective consists of conveying knowledge about how to encourage patients to take an active part in the decision-making process.

The online tutorial consists of four units: background of SDM (theoretical framework), presentation and explanation of the six SDM steps (illustrated by video clips established by the project team), the basic principles of risk communication in medicine, and the basic principles of communication skills. This tutorial system is integrated in an established learning platform (Moodle).

#### Control group (CG)

Physicians randomized to the CG receive no SDM intervention during the study period. However, as an incentive for study participation, they are offered access to the web-based SDM online training and receive a booklet summarizing all relevant information on SDM at the end of the study (after finishing T_1)_.

### Standardized Patients (SPs)

As it is difficult to include real patients in randomized controlled trial (RCT) interventions, we decided to use SPs. In German- and English-speaking countries, SPs often play a crucial role in the assessment of communication skills in medicine [49, 50]. SPs are mostly lay actors who are trained to simulate a real patient in a standardized way [51]. The standardization of the patient role allows comparison of the physicians SDM performance in a most effective way.

#### Training of the SPs

The role training for the SPs was conducted by an experienced professional trainer for actors who oversees the SP unit at the medical faculty in HD. In cooperation with oncologists, we developed three case vignettes for colon cancer (three male roles) and four for breast cancer (four female roles). The vignettes aim to represent an average cancer patient at an average diagnostic and treatment difficulty level for the physicians and to avoid special or difficult circumstances.

We will offer regular meetings for the SPs with the project team to discuss potential problems emerging during their encounters with the physicians. Furthermore, we want to ensure that no SP comes in personal conflict with his role as a cancer patient. Our professional trainer for actors will also stay in close contact with the SPs during the duration of the study and will give additional training lessons if needed. To reassure the standardization of the encounters and be able to intervene early when aberrations are obvious, the project team watches the videotaped consultation after the SP returns from the physician.

#### Procedure of the SP consultations

A member of the project team contacts the participating physician via telephone or e-mail to make an appointment for the consultation with the SP. A SP is then selected by availability and fit between diagnosis of case vignettes and the physicians’ field of work (colon or breast cancer). Care is taken to send different SPs with different case vignettes at T_0_ and T_1_ per physician. All physicians are aware that the consultation takes place with SPs and not with real patients. One week before the consultation the physician receives a letter including the medical information of the fictitious patient (e.g., size of the tumor, etc.). Furthermore, the physician receives information material about the available treatment options and also information about the recurrence probability of the particular disease (colon or breast cancer). This is to ensure basic knowledge also for less experienced physicians. The consultation takes place at the working place of the physician and is videotaped by the SP via a tablet computer. After the consultation, the SP gives out a paper questionnaire to the physician and completes his own questionnaire and subsequently brings the questionnaires as well as the tablet with the videotaped consultation back to the project team.

#### Recruitment of SPs

To provide the necessary amount of SP contacts, we need at least 12 SPs (six in each study center) who are recruited via notice boards and online via university web-sites. SPs must meet the following inclusion criteria:

- Age range 40 to 65 years for female (breast cancer vignettes) SPs and 50 to 75 years for male (colon cancer vignettes) SPs (relying on the approximate onset of the diseases)
- Fluent in German
- Flexible availability
- Mobility either by train or by car (costs will be refunded)
- Guaranteed participation for a period of at least 1.5 years
- No cancer disease in the own history or in the family

### Outcomes

**Fig. 2:**
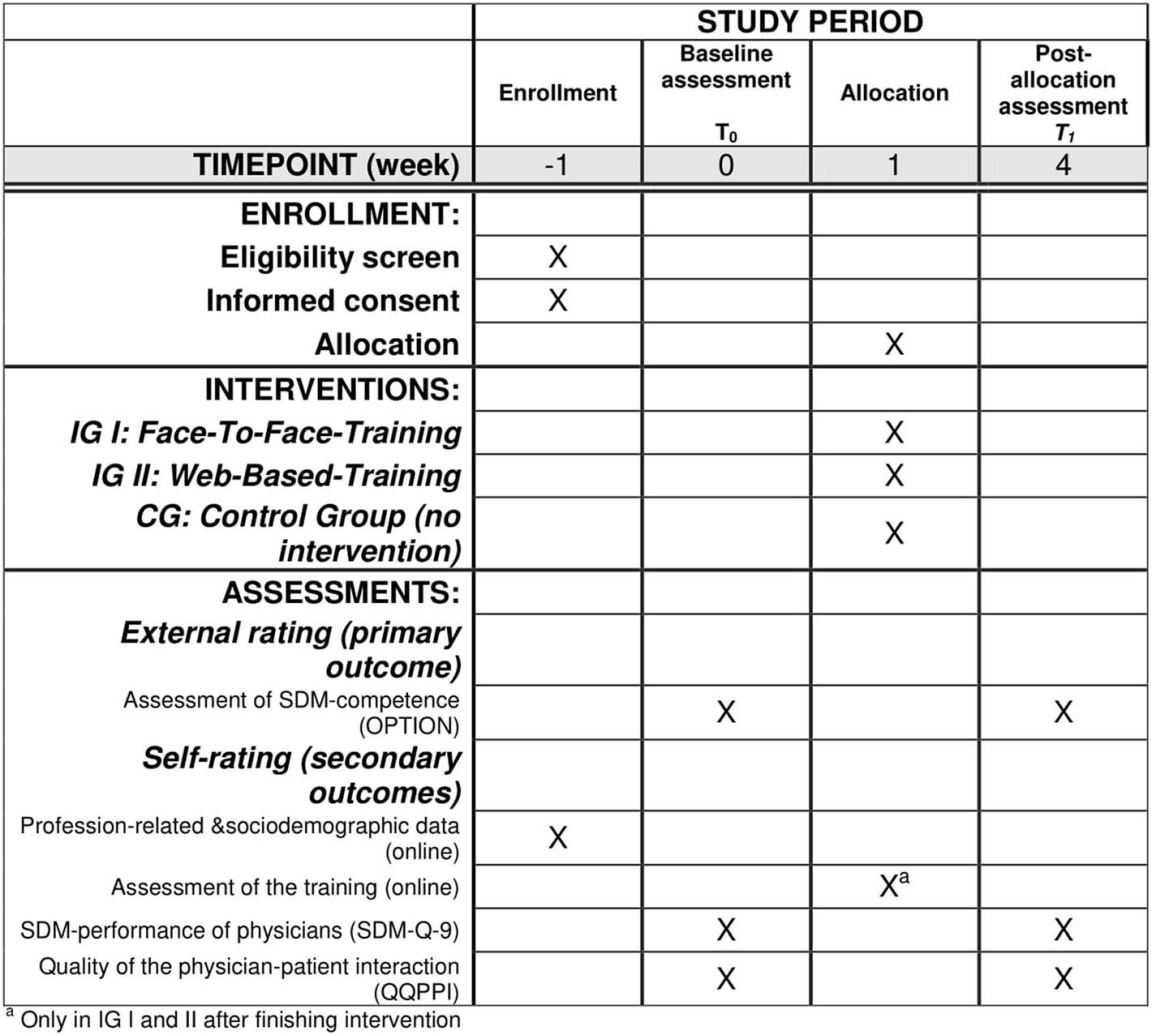
Schedule of enrollment, interventions, and assessments (Standard Protocol Items: Recommendations for Interventional Trials (SPIRIT) Figure)

Overall schedules of enrollment, interventions, and assessments can be found in Fig. 2.

#### Primary outcome

As primary outcome for *hypothesis 1* the German version of the OPTION-12 scale (Observing Patient Involvement 12; 36, 37, 38 References are not "active") is used. The OPTION-12 scale assesses the physicians’ SDM competence in consultations. The OPTION-12 scale is a well-established external, rater-based observing method for assessing the process of shared decision-making [52]. It has a good reliability with a value of 0.79. The consultations with the SPs are rated based on 12 criteria. Examples for each rating category per item will be determined beforehand. Each consultation is rated by two blinded and independent, well-trained raters using videotapes and transcriptions of the films. The mean value of both raters will be used. Furthermore, to describe interrater-reliability, Cohen’s Kappa will be computed.

#### Secondary outcomes

To test *hypothesis 2* we use the validated German version of the SDM-Q-9 (Fragebogen zur Partizipativen Entscheidungsfindung; Engl. Shared Decision Making Questionnaire-9 – Patient Version; [40, 53]). The SDM-Q-9 assesses SDM performance in nine items from the perspective of SPs. Furthermore, to test *hypothesis 3* we use the validated German version of the QQPPI (Fragebogen zur Arzt-Patienten-Interaktion (FAPI); Engl. Questionnaire on the Quality of Physician-Patient Interaction; [41]). The QQPPI assesses the quality of the physician-patient interaction from the patient’s perspective. The questionnaire includes 14 items related to adequate information transfer, involvement in decisions and the patient’s perception of being taken seriously by the physician. To test *hypothesis 4* regarding participating physicians’ satisfaction with the training we will use a self-developed training evaluation with 10 general items appropriate to both implementation strategies. This questionnaire is also used to control the treatment integrity and is implemented as an online questionnaire using the software SoSci Survey (www.soscisurvey.de); see for detailed description “Interventions.”

#### Further measurements

We assess sociodemographic data and profession-related variables (sex, work setting, years of professional experience) by a self-developed online questionnaire using the software Social Science Survey (SoSci Survey) (www.soscisurvey.de). Further measurements at different time points for physicians and SPs will be integrated in this study. However, because these outcomes are not the focus of the hypotheses of this study protocol, they will not be described further.

### Sample size

The sample size calculation is based on the main *hypothesis1* and the method of analysis. A review of the efficacy of non-web-based SDM interventions [54] including five RCTs described significant comparisons in two of the five studies. In the significant studies, effect sizes were calculated as *d* = 1.06 and *d* = 2.11. Taking the non-significant results into account, a conservative estimation of effect size seems appropriate. Further, according to a meta-analysis [26], web-based trainings for physicians achieved high-range effect sizes regarding skills (Hedges’ *g* = 0.85) and behaviors/effects on patient care (Hedges’ *g* = 0.82). Therefore, an effect size of *d* = 0.50 is expected for the primary outcome (SDM competence assessed by OPTION-12 at T_1_ adjusted for SDM competence at T_0_).

The estimation of the sample size for a covariance-analysis (ANCOVA) is comparable to an analysis of variance (ANOVA) focusing on the difference between adjusted means. A power analysis via GPower Version 3.1.2 [55] for an ANOVA (one factor with three groups: IG I, IG II, CG) was performed. The results show with a type-1 error of 5% a sample size of 159 physicians is needed to detect a statistically significant effect size of *d* = 0.50 with a power of 80%. Due to randomization issues, a sample size of 162 participants is targeted.

### Recruitment

Each study center will establish lists of physicians throughout Germany working with colon or breast cancer patients, either in cancer centers or as practitioners, by a systematic Internet research and using the lists of federal statutory physicians’ associations (Kassenärztliche Vereinigungen). We will subsequently send postal invitations to participate, provide flyers to each center’s responding practitioner and have a reference on our webpage that provides detailed information about the study. If e-mail addresses are available, we will send the invitation for participation in a digital format. Furthermore, if preferred (e.g., from a head of a cancer center), we will attend the cancer center and introduce the study and the corresponding SDM training program personally. Interested physicians are asked to contact the project team via e-mail or telephone. The responsible project team subsequently sends an e-mail including the link for the first online questionnaire to the participant and allocates a code to each participant in a consecutive numerical order.

## Methods: Assignment of interventions

### Sequence generation

Randomization will be conducted by a computer-generated block-randomization performed by the open source randomization software Randi3 [56]. The randomization is stratified by study center and blocked into three randomization blocks (per study center) with 27 participants (nine per IG I, II and CG) to guarantee equal sample sizes in each of the three groups when targeted sample size is not achieved (2 study center × 3 randomization blocks × 3 groups × 9 participants = 162 participants).

### Allocation concealment mechanism

The code of the participating physicians will be randomized after the first consultation with the SP (T_0_). For this, both study centers send the code for randomization to a study nurse per e-mail, who conducts the computer-generated randomization and informs the study centers about the result per e-mail.

### Implementation

The project team provides the result of the randomization to the participant by e-mail and coordinates the next steps in accordance to the result. For participants randomized to IG I, an appointment for the face-to-face-training is arranged, for participants of the IG II and CG an appointment for T_1_ is arranged and additionally for participants of IG II a link for the web-based interactive SDM online training is sent per e-mail.

### Blinding

Due to the nature of this trial, blinding of the physicians is not feasible. However, SPs and the independent raters, when rating the videotaped and transcribed consultations, are blinded regarding the group (IG I, IG II, CG) and the point of measurement (T_0_, T_1_).

## Methods: Data collection, management, and analysis

### Data collection methods

Before we include participants we will assess sociodemographic and profession-related data (medical specialization, years of professional experience) through the online survey for participating physicians. After a consultation with the SP, both the SP and the physician obtain a set of paper-based questionnaires (in T_0_ and T_1_). Consultations between the physician and the SP are recorded on video (by the SPs) and subsequently assessed by the research team. The evaluation of the intervention is conducted with an online survey after finishing either face-to-face (IG I) or online-training (IG II). In the case that participants fail to complete an online questionnaire, the project team sends a reminder via e-mail.

### Data management

Data management occurs in the study centers, and each center is responsible for its own data. Data entry will be conducted by study assistants. We aim to conduct reciprocal random checks on the quality of data entry. At the end of data collection, the data sets will be pooled for final analyses.

### Statistical methods

#### Statistical analyses

To test the main *hypothesis 1* an ANCOVA is conducted. The mean of the OPTION-12 at T_1_ serves as the dependent variable and the group factor (IG I, IG II, CG) serves as independent variable. The mean of the OPTION-12 at T_0_ serves as a covariate. Post-hoc tests using a Bonferroni correction will be conducted to analyze single contrasts. To test the secondary *hypotheses 2 and 3*, two ANCOVAS and post-hoc tests, comparable to the ANCOVA for *hypothesis 1*, will be conducted with the means of the SDM-Q-9 and QQPPI at T_1_ as dependent variable, the group as independent variable and the means of SDM-Q-9 and QQPPI at T_0_ as covariates. To test *hypothesis 4* regarding the satisfaction a *t* test for independent groups with IG I and II as independent variable and the mean of the 10 general items of the training evaluation as dependent variable will be conducted. For data management and analyzing, SPSS© will be used.

#### Drop-outs and missing values

Participants, who drop out after T_0_, are considered for the main outcome (OPTION-12, no missing values at T_0_ due to inclusion/exclusion criteria II and nature of external rating) according to the intention-to-treat principle using a single imputation method, in more detail we impute the mean difference of other group (MOTH-D) [57]. Therefore, the mean difference (per item) between T_1_ and T_0_ of available cases in CG is added to T_0_ of drop-outs in IG I and II and vice versa the mean difference (per item) in IG I and II is added to T_0_ of drop-outs in CG. Additionally, an analysis with available cases which completed the study (analysis per protocol) will be reported in contrast.

Missing data in the ratings of the SPs (SDM-Q-9 and QQPPI; secondary outcome) due to drop-outs will be handled like the main outcome using MOTH-D. Additionally if missing data in a single item occurs in more than 50% of all questionnaires (T_0_ and T_1_) of available cases, this item will be deleted for all SP ratings because this item does not fit into the nature of consultations in this study. It is unlikely that missing values will emerge to a greater extent as SPs were reminded frequently to complete the questionnaire completely. However, single missing values in the SDM-Q-9 and QQPPI will be not imputed. Instead, a mean value is calculated with the remaining completed items of the questionnaire.

For analyzing the training evaluation missing cases will be imputed by the worst possible mean. An analysis with available cases will be conducted and compared.

## Methods: Monitoring

### Data monitoring and auditing

The Data Monitoring Committee (DMC) consists of KG, NM, and ChB in Heidelberg and SD, CB, and MH in Hamburg. For each of the two recruiting centers reports of data recruitment status will be assessed in a written report every 3 months by KG and SD and mailed around to the whole study group. Regular telephone conferences every 3 months with all study group members participating will serve to discuss improvement strategies and initiate adjustment strategies in case that recruitment is delayed.

The DMC is independent of the sponsor. However, the sponsor (DKH) is controlling for progress in recruitment by asking for an interim report after the first 2 years of the grant, and will only grant finances for the third and last year of the study if recruitment is progressing according to plan. Therefore, the sponsor can make the final decision to stop the trial early after 2 years in case that milestones are not met by that time point.

### Harms, confidentiality and access to data

There are no harms expected for the participating physicians. SPs will be supervised by members of the project team; relevant adverse events are unlikely. The data protection provisions of the Federal Data Protection Act are conformed to in the implementation of the study. The survey cannot be conducted completely anonymously, due to the videotaped consultations and the repeated survey that is required for subsequent analysis. However, data will be pseudonymized. Subsequent assignment of data to a person is only possible by means of randomized codes. The transcripts for each video recording will also be anonymized and identified by the appropriate code.

All information and videotaped consultations will be kept strictly confidential and no personal data or videotaped consultation will be passed to third parties. In case of the publication of results, it will be ensured that no inference can be made about the identity of participants. Personal data will be deleted either at the end of the study or, at the latest, after 5 years.

## Discussion

This trial will assess the effectiveness and acceptability of two innovative SDM training programs for physicians. We aim to analyze which improvements in SDM competence can be achieved by these training programs and their impact on the physician-patient interaction and to test if the dissemination methods differ regarding their efficacy.

Although we aim to meet the needs of the physicians in their daily routine, we anticipate some challenges in recruitment for the study. As the training is voluntary, it cannot be assumed that it can be performed during working hours. If the participants must complete the training in their leisure time, this could likely influence their motivation and, therefore, the expected effects of the training. Furthermore, face-to-face SDM training will be conducted by different members of the project team. Although we intend to standardize the coaching sessions, as far as possible, it cannot be avoided that there will be some differences between training lessons performed by different trainers. Although we train the SPs in oncological case vignettes to enable them to act in a standardized way, every SP brings his own personality and attitudes to the consultations. Furthermore, it is inevitable that the SPs will undergo an increase in medical knowledge as the study evolves and, therefore, may show a behavior change during later consultations. We will try to compensate for this by strictly monitoring consultations, and we will give additional training lessons if needed. We expect in IG I with face-to-face training to gain a higher level of satisfaction. However, for long-term implementation of the training, we assume that regarding the temporal expense an online tutorial is more feasible in daily routine compared to face-to-face coaching lessons. Despite these challenges, we also see several opportunities. Specifically, we consider our web-based training as an adequate and cost-effective way to convey SDM-related knowledge and competences. If this hypothesis is validated, then our training indicates that this training format should be extended to other medical areas. Furthermore, we consider our training to be an appropriate way to convey other communication-related challenges (e.g., breaking bad news) in daily medical practice.

## Trial status

Physician recruitment for the trial started in the fall of 2016 and will last until December 2018.

## Additional file

Additional file 1: Standard Protocol Items: Recommendations for Interventional Trials (SPIRIT) 2013 Checklist: recommended items to address in a clinical trial protocol and related documents. (DOC 120 kb)

## Abbreviations

ANCOVA: Analysis of covariance
CG: Control group
DKH: Deutsche Krebshilfe (German Cancer Aid)
ICH-GCP: International Conference of Clinical Harmonization of technical requirements for registration of pharmaceuticals for human use – Good Clinical Practice
IG: Intervention group
QQPPI: Questionnaire on the Quality of Physician-Patient Interaction (FAPI: Fragebogen zur Arzt-Patienten-Interaktion)
RCT: Randomized controlled trial
SDM: Shared decision-making
SDM-Q-9: Shared Decision Making Questionnaire-9 – Patient Version
SoSci Survey: Social Science Survey
SP: Standardized Patient
